# Integrated decision-making about housing, energy and wellbeing: a qualitative system dynamics model

**DOI:** 10.1186/s12940-016-0098-z

**Published:** 2016-03-08

**Authors:** Alexandra Macmillan, Michael Davies, Clive Shrubsole, Naomi Luxford, Neil May, Lai Fong Chiu, Evelina Trutnevyte, Yekatherina Bobrova, Zaid Chalabi

**Affiliations:** Complex Built Environment Systems (CBES), UCL Institute for Environmental Design and Engineering, UCL, London, UK; UCL Energy Institute, The Bartlett, UCL, London, UK; Department of Social and Environmental Health Research, LSHTM, London, UK

## Abstract

**Background:**

The UK government has an ambitious goal to reduce carbon emissions from the housing stock through energy efficiency improvements. This single policy goal is a strong driver for change in the housing system, but comes with positive and negative “unintended consequences” across a broad range of outcomes for health, equity and environmental sustainability. The resulting policies are also already experiencing under-performance through a failure to consider housing as a complex system.

This research aimed to move from considering disparate objectives of housing policies in isolation to mapping the links between environmental, economic, social and health outcomes as a complex system. We aimed to support a broad range of housing policy stakeholders to improve their understanding of housing as a complex system through a collaborative learning process.

**Methods:**

We used participatory system dynamics modelling to develop a qualitative causal theory linking housing, energy and wellbeing. Qualitative interviews were followed by two interactive workshops to develop the model, involving representatives from national and local government, housing industries, non-government organisations, communities and academia.

**Results:**

More than 50 stakeholders from 37 organisations participated. The process resulted in a shared understanding of wellbeing as it relates to housing; an agreed set of criteria against which to assess to future policy options; and a comprehensive set of causal loop diagrams describing the housing, energy and wellbeing system. The causal loop diagrams cover seven interconnected themes: community connection and quality of neighbourhoods; energy efficiency and climate change; fuel poverty and indoor temperature; household crowding; housing affordability; land ownership, value and development patterns; and ventilation and indoor air pollution.

**Conclusions:**

The collaborative learning process and the model have been useful for shifting the thinking of a wide range of housing stakeholders towards a more integrated approach to housing. The qualitative model has begun to improve the assessment of future policy options across a broad range of outcomes. Future work is needed to validate the model and increase its utility through computer simulation incorporating best quality data and evidence. Combining system dynamics modelling with other methods for weighing up policy options, as well as methods to support shifts in the conceptual frameworks underpinning policy, will be necessary to achieve shared housing goals across physical, mental, environmental, economic and social wellbeing.

**Electronic supplementary material:**

The online version of this article (doi:10.1186/s12940-016-0098-z) contains supplementary material, which is available to authorized users.

## Background

In the UK, much attention has been given to policies aimed at reducing carbon emissions from the housing stock as part of the UK's legislative commitment to achieve an 80 % reduction in greenhouse gas (GHG) emissions by 2050 [1]. Houses contributed a quarter of the UK’s total GHGs in 2009 [2]. It has been argued that effective policies and technologies already exist to achieve significant reductions [2] and successive governments have considered improving the energy efficiency of the housing stock to be one of the easier ways to achieve the large GHG emission reductions that are now urgently needed. Under current plans, the UK government has set out pathways that will see more than 14 million existing homes retrofitted to make them more energy efficient by 2020 [3]. However, retrofitting will not be successful without integrating physical changes with changes in people’s interaction with their homes [4].

Furthermore, the complexity of the housing stock; the importance of homes to people’s lives; and the wide spectrum of agents responsible for changes to houses all make housing an important area of “policy resistance” [5]. By this, we mean that policies may fail to achieve their intended objective, or even worsen desired outcomes, because of limitations in our understanding of housing as a dynamically complex system from policy design through to implementation. Unintended consequences across a range of possible outcomes for human wellbeing are also a substantial risk [6, 7]. This has been further demonstrated by Sabel et al. through their models of climate policy for seven cities in this issue [8]. Apart from the direct physical effects of temperature on health, housing design, availability and cost all have complex relationships with a wide range of public health outcomes. The full extent of these outcomes has been incompletely considered in previous integrated assessments of housing policy [9]. Separate to the agenda of decarbonisation, other government sectors are explicitly attempting to achieve other (and sometimes contradictory) goals around housing. These include reducing fuel poverty; improving housing affordability; using housing construction and the property market to stimulate economic growth; and reducing health inequities through housing interventions. A recent report from the All Party Group for Excellence in the Built Environment (*Re-energising the green agenda*) [3] highlighted a lack of integration across government departments and conflicting objectives as significant barriers to progress.

For these reasons, new approaches are needed to support decision-making about housing. Research across disciplines of urban policy-making for health, equity and sustainability suggests that these methods will need to: integrate the qualitative and quantitative knowledge held by different groups across policy, society and academia (*transdisciplinarity*) in a collaborative learning process; support decision-making through understanding complex systems; and explore the impacts of policies on a more integrated set of outcomes (e.g. health, environment, economy, social equity) [6, 7, 10, 11]. In this issue, Rietveld and colleagues demonstrate how utilizing these principles can successfully improve outcomes in the complex area of urban water and health [12].

In this paper we report on early policy-oriented research to develop a collaborative understanding of the complex system linking housing, energy and wellbeing. We used the principles described above to guide the research. In partnership with government, non-government, industry, community, and academic stakeholders, we aimed to identify a set of shared wellbeing outcomes across policies about housing in the UK; develop a set of criteria for assessing future policies; build a qualitative understanding of the dynamic system structure; and begin to assess and identify policies that might effectively optimize shared goals while minimizing undesirable impacts.

## Methods

We used participatory system dynamics modelling (SDM) [13–15] to involve industry, community, academic and policy stakeholders in a process that explored the dynamic effects of realistic policies in the UK. SDM is built on the following underlying characteristics of complex systems [16]:

1. They include many interacting variables that change over time
2. It is this pattern of interaction that is a key driver of system behaviour over time
3. Interaction between variables is characterized by reinforcing loops, which amplify dynamic system patterns of behaviour and balancing feedback loops
4. Complex systems are also characterized by the accumulation of “stocks” that could include people, information, or material resources
5. Time is an important component of complex systems and the pattern of cause and effect relationships may change variables at different rates over time, creating tensions between short- and long-term policy effects

Saaed [17] describes a useful generalisable heuristic for an SDM process that uses iteration to move from desired outcomes through understanding of problems related to these outcomes, qualitative representation of the system structure, development of a dynamic simulation model, scenario experimentation and policy design. A SD simulation model consists of a set of differential equations whose solutions are approximated to demonstrate dynamic system behavior, enabling trajectories over time in outcomes of interest to be explored and compared for future policy options. While we consider experimentation using a dynamic simulation model a crucial step towards developing a robust system understanding and elucidating the consequences of policy interventions, this paper describes the first part of the heuristic, namely the development of an initial shared qualitative system understanding of housing, energy and wellbeing.

System dynamics modelling (SDM) enables a more complete and dynamic causal understanding that accounts for the five complex system characteristics above. In addition, SDM enables dynamic simulation to explore the effects of proposed policies over a chosen time scale. SDM (with varying degrees of participation) has been successfully used to improve decision-making in a variety of disciplines, including energy planning [18, 19]; policy-making about housing markets [20, 21]; uptake of energy efficiency in housing [22, 23] and urban transport and land use planning [24, 25]. As with most SDM efforts, these examples aimed to provide insights about the dynamic effects of policy alternatives by relating them to the system structure, rather than attempting to make precise absolute predictions about future outcomes, something that is not possible in these contexts.

In this research we used a combination of primary and secondary data to develop a qualitative set of feedback loops, known as causal loop diagrams (CLD), to describe a shared dynamic causal theory about the relationships between housing, energy and wellbeing. We took the view that the construction of such CLDs is akin to the development of a constructivist grounded theory described by Charmaz [26] and oriented our primary data analysis accordingly to be primarily inductive; include both semantic and latent ideas and assumptions; and consider individual accounts to be manifestations of the underlying sociocultural and built environmental structures which were the subject of our research [27].

**Fig. 1:**
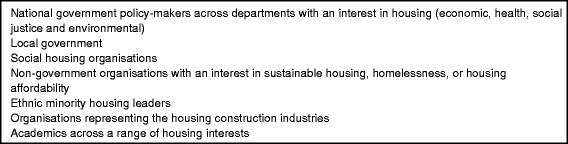
*A priori* sampling frame used to identify representatives

We used a purposive sampling strategy based on an *a priori* sampling frame to identify government, industry, community and academic groups with an interest in policies about UK housing (see Fig. 1), aiming for a group of approximately 30 representatives [28]. Initial contact with stakeholders was also opportunistic, since the research team knew many stakeholders who fitted the sampling frame. We considered it important to include organisations with a range of different interests in housing, but also a hierarchical range of representatives. In keeping with recent stakeholder theory across disciplines [29–31], we aimed to include representatives with the power to influence government policies about housing, those who could implement decisions, those whose perspectives are important but rarely heard (for example low-income households), as well as a range of values and political ideologies. Some participants represented named organisations, while others were part of more abstract categories of actors (for example “social housing providers”). We recruited participants by direct contact with pre-determined groups, as well as via the networks of the researchers and established participants. The process of recruitment continued throughout the project as relationships were built with new organisations and the group’s understanding of the system and problem situation evolved [29].

We undertook individual semi-structured interviews with participants. We used a single opening question: *What do you think are the links between houses and the wellbeing of individuals, families and communities in the UK?* For each link identified, further probing questions were asked:

- *Let’s talk a bit more about the causes of this – why has/does this occur/ed?*
- *Let’s talk some more about the consequences – what happens because of this?*

During the interview we used cognitive mapping [32] to make explicit the participant’s internal understanding of the complex connections between housing, energy and wellbeing. Cognitive mapping is one technique for exploring mental processes, particularly when the relationships between causes and consequences are of interest, as well as considering opposing choices or behaviours [33]. Furthermore, cognitive maps have been identified as a useful starting point for collating and comparing the views of a number of stakeholders in relation to a policy issue [34]. A cognitive map comprises concepts linked by arrows demonstrating polarity to form a chain of underlying causes and consequences. In addition, interviews were digitally recorded and partially transcribed. At the end of each interview, participants were asked to list and then prioritise a set of criteria against which policies about housing should be measured (policy assessment criteria).

The cognitive maps were digitalized using Decision Explorer® (Banxia Software). These were returned to interviewees for review and their comments were used to clarify and refine the individual maps. We undertook a thematic analysis of the interview recordings and cognitive maps together. Although we brought to the analysis our own underlying mental models of public health, wellbeing and energy use in housing, we undertook a primarily inductive analysis of the variables and relationships discussed in the interviews, without an *a priori* coding frame. A single coder undertook initial coding of variables. This was followed by discussion of the codes and potential themes among members of the research team. The themes were then used to re-code the variables in two iterations between researchers. The prevalence of each code and theme was recorded across the whole dataset, and these were used to assist with understanding the prominence of codes and themes in the interviews, acknowledging that prevalence reflected the make-up of the participant group as well as the dominance of themes in the interviews.

The thematic analysis and digital maps were triangulated with our own previously reported cross-disciplinary literature review, which linked policies to decarbonize housing with broad wellbeing outcomes across “buildings, people and nature” [7]. Together these data were developed into an initial set of CLDs using Vensim (Ventana Systems) system dynamics software. The set of CLDs was divided into the themes emerging from the thematic analysis of interviews. In developing the CLDs we were careful to identify and maintain opposing or contradictory theories between participants, by including these competing theories in the same diagram for review, discussion and evidence-gathering.

A subsequent stakeholder workshop involved introducing system dynamics modeling to the participants and mixed small group work to review and refine the draft CLDs. Following the workshop, further responses was elicited, particularly from stakeholders who were not present at the workshop. In addition, and where possible, contradictory theories were discussed and, where data were readily available, some theories could be discarded in a collaborative learning environment. A working version of the CLDs was then circulated to all the participating organisations. All stakeholders were invited to a second workshop where participants were provided with opportunities to develop early policy recommendations from their collaborative learning and practice and use the CLDs to consider realistic policy proposals.

In preparation for future policy assessments, the list of assessment criteria elicited during the interviews was developed further in a participatory manner [35]. In order to create a manageable prioritised list, the top five policy assessment criteria from each interview were combined to develop a complete draft set of criteria. Very similar or identical criteria were grouped together and counts were made of the number of participants identifying each criterion and the rankings they allocated. All criteria were then grouped into those that were identified as priority one by at least one participant; criteria that were ranked in the top five; and those that were not in any participant’s group of five top criteria. The contents of this initial list were refined to develop a set of criteria that were, as much as possible, mutually independent; able to assess the differences between policies (i.e. having values that are likely to vary between policies); and eliminate criteria that were either composites of others, policy options themselves or overarching goals (e.g. human wellbeing). Final names, definitions and possible indicators for each criterion were then developed. Criteria that were ranked first or second by at least one participant were put forward as candidates for the final shared list. We used a silent negotiation procedure at the first workshop previously described to develop a consensus set of policy assessment criteria [36, 37]. Based on this negotiation, an initial set of nine criteria was proposed.

**Fig. 2:**
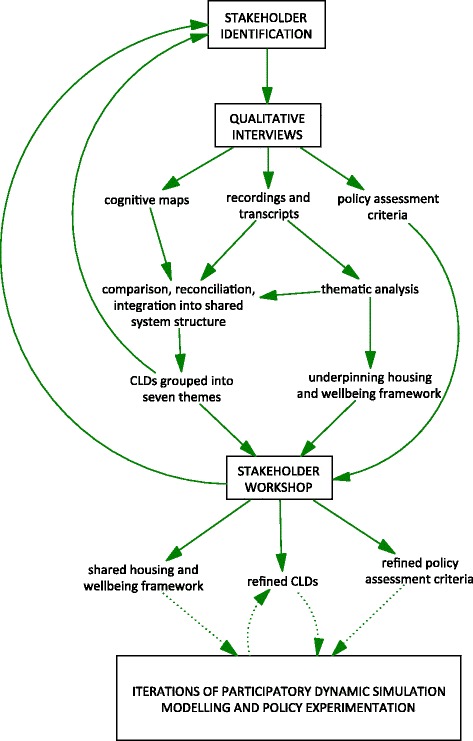
Summary of model development process

The steps described above to develop and refine the qualitative SDM are summarised in Fig. 2.

The research was exempt from requiring formal ethics approval by the University College London ethics committee because it involved non-vulnerable and public arena participants in non-sensitive research procedures (http://ethics.grad.ucl.ac.uk/exemptions.php). All participants were provided with an information sheet and took part voluntarily, having signed a consent form.

## Results

### Participants

We approached a total of 52 organisations and agencies. Over 50 stakeholders were recruited, representing 37 organisations. These included six national government departments; five representatives from local government; 14 non-government organisations; a group of six minority-ethnicity housing leaders (community roots group); five industry organisations; and eight academic institutions. Some stakeholders represented more than one sector. Different members of the stakeholder group were represented during the interview phase and at the workshops. The organisations participating at each stage have been mapped to demonstrate the level of participation and change in participants over time (Additional file 1).

Sustained effort was required over a longer period to identify and recruit minority ethnicity housing activists. This group of six participants came together following the first workshop.

We interviewed 33 participants across national and local government, non-government organisations, construction and housing industries and academic research. Twenty-six stakeholders took part in the first workshop and an overlapping group of 26 participated in the second workshop. In between, two smaller meetings were convened to gain the specific input of the community roots group, which were attended by six and five members, respectively.

### Shared connections between housing, energy and wellbeing

Ten main themes were identified as a result of the thematic analysis. These are described, along with their sub-themes, in Table 1, which also describes how often these themes were identified across all the interviews (“prevalence”). The themes covered aspects of the physical nature of houses; how houses are put together to develop communities and in the context of other land uses; the relationships between housing and wider systems such as demographics, urban planning, property and labour markets; and the influence of these on the participants constructions of wellbeing as a notion. The most commonly and deeply discussed theme related to influences on the energy efficiency of houses. The dominance of this theme reflects the current UK policy focus on housing, and was perhaps unsurprising given the participants’ knowledge about the provenance of the research. Perhaps more surprising was the importance participants placed on neighbourhoods and social wellbeing, which was the second most commonly discussed theme from the interviews.

**Table 1:** Summary of themes resulting from the thematic analysis

Themes and subthemes	Number of variables in theme	Prevalence across the dataset
Indoor temperature	69	268
Heating and fuel poverty	53	203
Need for cooling	6	32
Outdoor ambient temperatures	5	21
Thermal comfort	5	12
Air quality and ventilation	27	179
Ventilation	13	70
Moisture and damp	4	63
Chemical exposures	10	46
Overcrowding	21	117
Neighbourhoods	67	452
Community social connection	46	311
Sense of security from crime	11	94
Tenure security	10	47
Energy use and efficiency	136	503
Influences on the energy efficiency of houses	91	362
Energy supply and pricing	27	94
Transport energy use	18	47
Housing quality	83	213
Influences on building quality	78	201
Exposure to light	5	12
Demographic change	27	100
Adaptation of housing to climate change	47	174
Land use and urban planning	60	170
Aspects of wellbeing related to housing	48	279
Overall health and wellbeing	10	52
Mental health and emotional wellbeing	14	107
Physical health	17	95
Economic wellbeing	5	15
Environmental wellbeing/sustainability	5	10

The following different specific aspects of what could be considered overall human wellbeing emerged from a thematic analysis of the interviews:

1. Social and cultural wellbeing and community connection
2. Physical health
3. Mental health, homeliness and happiness, stress
4. Local economic thriving, household income and employment, a stable economy
5. Adaptation and mitigation of climate change
6. Sustainable resource use

These aspects of wellbeing were used as an underpinning framework for the workshops, as well as for considering the objectives of housing policy.

In describing the relationships between housing and wellbeing almost all the representatives implicitly held a view of wellbeing that privileged the wider structural influences (for example at a policy, economy, societal and built environment level) on people’s lives rather than “lifestyle” or individual choices (agency). On the other hand, there were discussions about how previous and current attempts to intervene (for example through the Code for Sustainable Homes, or other historical housing improvement programmes) had been less successful than hoped at improving people’s lives or reducing energy use. Participants who discussed the impacts of housing energy use on climate change, as well as other environmental impacts of housing, did so within the context of discussions about housing and human wellbeing, suggesting they implicitly considered environmental sustainability to be one aspect of human wellbeing.

Almost all the representatives we interviewed emphasised the need for the aspects of wellbeing listed above to be fairly distributed across different groups, including by income, ethnicity and generation (or life-stage), and that housing was an important contributor to existing wellbeing inequalities, and furthermore was a factor that could be modified.

### Overview of the causal loop diagrams (CLDs)

Although we were able to establish themes from the interviews, the interview cognitive maps made it clear that these themes were all deeply intertwined. The thematic analysis and the cognitive maps were together used to guide the development of the initial CLDs, which were then refined during and between the subsequent workshops and meetings.

The causal maps represent interactions between variables (e.g. things, actions, feelings) that are likely to explain observed trends in the housing, energy and wellbeing “system”. Some of these variables are levels that we are interested in measuring over time (“stocks”), while others are rates (or “flows”) that affect these levels. The variables are connected by causal links (arrows), and together form feedback loops – cycles of cause and effect that determine how a system behaves and changes over time. There are two kinds of feedback loop: reinforcing loops (R), so named because over time they reinforce patterns of system behaviour; and balancing loops (B) that can dampen and limit trends over time.

**Fig. 3:**
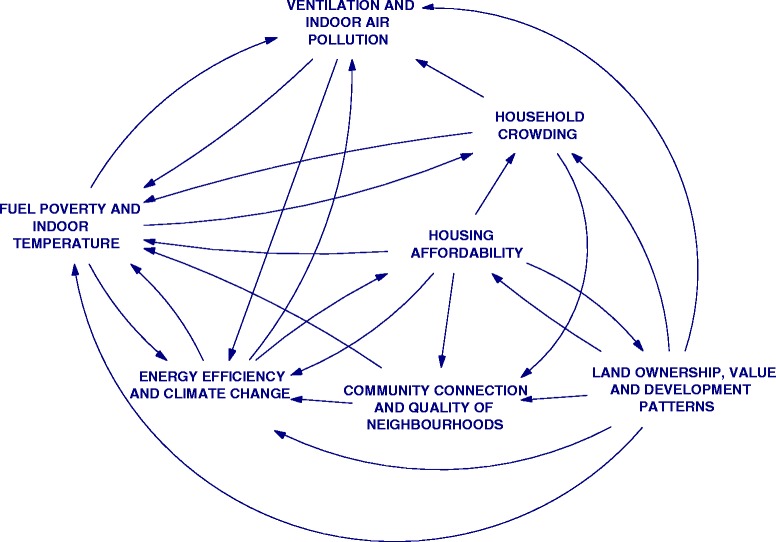
Overview of the seven themes used to organise the housing, energy and wellbeing CLDs

Of the ten themes that were elicited from the interviews, the aspects of wellbeing and demographic themes were spread across all other themes in the CLDs. Further, the interview cognitive maps and workshop discussions demonstrated that housing quality and patterns of land use were closely linked. They were therefore included in a single CLD. This left seven interconnected themes, which were used to organize the CLDs. An overview of these themes and their connections is provided in Fig. 3. A single example of one of the CLDs is provided in the next section. The full set of CLDs is described on the research project website: http://www.bartlett.ucl.ac.uk/iede/research/project-directory/projects/housing-energy-wellbeing.

### Community connection and the physical quality of neighbourhoods

Stakeholders considered that the physical quality of neighbourhoods and community social connection at the neighbourhood level were particularly important for wellbeing, as well as influencing a range of other housing objectives. These other objectives included energy efficiency and energy supply; adaptation to climate change; tenure security; land development patterns and the physical quality of houses. Local social connection was considered to be one of the important outcomes of policies about housing and is therefore shown as a stock. Because stakeholders discussed this type of connection as contributing positively to wellbeing, it could be seen as equivalent to the bridging social connection described in the literature [38] – connections between people who aren’t necessarily alike, to enable acting together for the common good. There was agreement among stakeholders that this stock had been declining over time. Furthermore, there was a shared desire to turn this trend around with beneficial effects for wellbeing (e.g. through social support, local physical activity and less crime) and energy use (e.g. through less travel for social connection, greater community capacity to support energy interventions). The concept of “quality” as it relates to housing and neighbourhoods has not been clearly defined, although stakeholders tended to describe physical aspects of the neighbourhoods and houses including levels of maintenance; usable green and shared spaces; attractive local places for people to meet; and safe places for children to play. Aspects of “beauty” relating to housing and neighbourhoods were also discussed in relation to the notion of quality. On the other hand, litter, graffiti, neglected buildings and public spaces were all considered to detract from neighbourhood physical quality. It was considered important by some participants that the residents of a neighbourhood should define “quality” themselves.

**Fig. 4:**
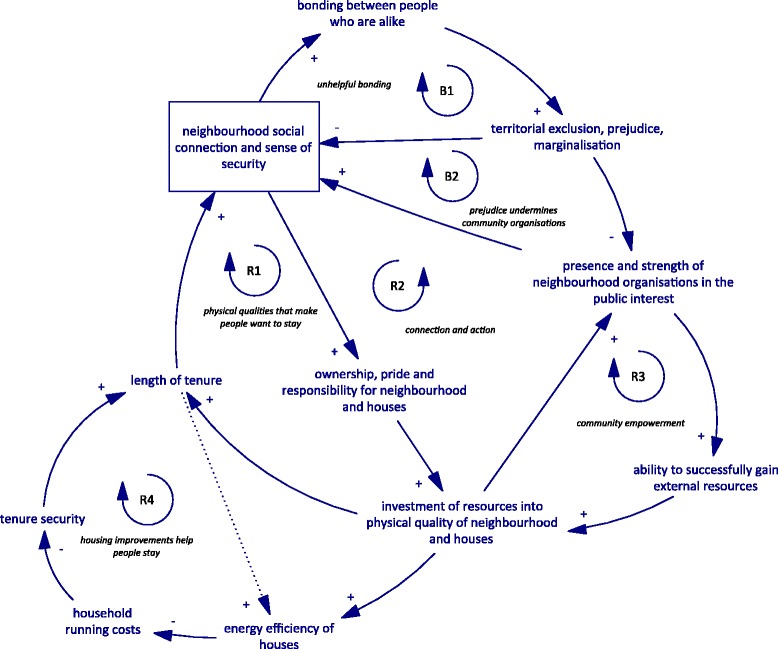
Community connection and the physical quality of neighbourhoods. Arrows with a positive sign (+) indicate a change in the variable at the arrow-tail leads to a change in the variable at the arrow-head in the same direction. Arrows with a negative (−) sign indicate a change in the arrow-tail variable leads to an inverse change in the arrow-head variable (opposite direction). R – Reinforcing loop, the result of which is an amplification of the initial pattern of behaviour. B – Balancing loop, the result of which may be to dampen the initial pattern of behaviour or create oscillation). The dashed connection was one where there remained disagreement about the relationship

The relationships in this CLD were considered by stakeholders to be currently dominated by reinforcing loops. While some are helpful for improving wellbeing and patterns of energy use, others serve to entrench poverty and poor social wellbeing. The CLD is provided in Fig. 4, with a description of the feedback loops below.

*R1 physical qualities that make people want to stay*: it was suggested that greater social connection and sense of security from crime leads to greater ownership, pride and sense of responsibility by residents. This leads to greater investment of resources by residents, landlords and local government into the physical aspects of houses and neighbourhoods. Improved houses and neighbourhoods (including amenities, green spaces and other places for locals to meet) makes people want to stay longer, increasing social connection and sense of security. Existing research about social connection supports these links (see for example [39, 40]).

*R2 connection and action*: Stakeholders proposed that improving the physical quality of neighbourhoods (including quality of green space and “third spaces” or other places where locals could meet) leads to stronger and more numerous neighbourhood-level social connections – either directly or through longer tenure. In turn, these connections can enhance community capacity to take action in the neighbourhood by strengthening local organisations that act in the public interest. Stronger and truly representative organisations in turn further enhance neighbourhood social connection. There was some disagreement about how successfully the design of physical spaces could be used to influence social wellbeing in this way.

*R3 community empowerment*: the strengthening of local public interest organisations through improvements to the physical quality of neighbourhoods was also considered to lead to greater ability of these organisations to attract external funding and other resources, enabling further improvements to the physical environment.

*R4 housing improvements help people stay*: as well as making residents want to stay in an area, improvements to houses (including energy efficiency improvements) may reduce household running costs and improve tenure security, allowing people to stay longer and further enhancing neighbourhood social connection and the investment of resources into improvement. There was disagreement about a more direct link between tenure security and the energy efficiency of housing (either through investment or behaviour).

There are two balancing loops that represent limits to the positive impacts of increasing social capital – in other words the potential negative effects of “too much” social capital, or when bonds between people who are very alike do not contribute positively to the public interest.

*B1 unhelpful bonding*: increasing social capital can lead to stronger bonds between people who are alike in ethnicity or socioeconomic status. In turn this can lead to territorial exclusion, prejudice and marginalisation of other groups. This can then undermine further improvements in local social connection and sense of security. Similarly, these same patterns of increasing social capital, exclusion and marginalisation can also undermine neighbourhood organisations (*B2 prejudice undermines community organisations*).

Neighbourhood social connection was considered to have varying importance by life stage, being particularly important for children and older people. It was argued, though, that neighbourhoods that successfully encouraged this kind of social connection would allow people to continue to live in neighbourhoods of their choice at different life stages. There was some debate about how community level income, ethnic and age mix fed into these community connection loops. While some argued that diversity would support community connection, resilience and positive action, others suggested that “super-diversity”, particularly when accompanied by short tenures and in the absence of resources, was not conducive to positive local community connection.

### Shared policy assessment criteria

Due to a large number of participants in the workshop, the silent negotiations procedure was conducted independently by three smaller groups. Participants were mixed by main role (policy, industry, community/non-government organisation, academic). Three negotiated lists of criteria resulted (Table 2). These were voted on at the end of the procedure, resulting in a final consensus list of nine policy assessment criteria, highlighted in Table 2.

**Table 2:** Results of the silent negotiation exercise to determine shared policy assessment criteria (the final preferred list is highlighted)

Policy criteria	List B	List C
List A (preferred list)		
Carbon emissions from housing	Carbon emissions from housing	Carbon emissions from housing
Community connection	Community connection	Community connection
Fuel poverty	Fuel poverty	Employment
Housing adaptation to climate change	Green space and neighbourhood	Fuel poverty
Housing affordability	Housing affordability	Green spaces and neighbourhood quality
Mental & emotional wellbeing	Mental	Housing adaptation to climate change
Physical wellbeing/health	Physical wellbeing/health	Housing affordability
Policy coherence	Social and income equity	
Social and income equity		Physical wellbeing/health

## Discussion

### Principal findings

Using participatory system dynamics modelling we have successfully brought together a broad range of industry, policy, community and academic stakeholders in the area of UK housing; established a wellbeing framework for considering policies about housing that incorporates physical, mental, environmental, social and economic wellbeing; collaboratively developed an initial complex qualitative system dynamics model made up of seven sectors; and identified a shared set of criteria against which to measure and compare future proposed policies about housing, regardless of the primary objective of those policies. Previous studies have used system dynamics modelling to understand specific parts of the housing system, particularly housing markets, construction and affordability. In addition, there have been other studies which have assessed some of the health consequences of policies to reduce greenhouse gas emissions from housing. However, this is the first comprehensive model of the housing system linking shared objectives for human wellbeing.

The collaborative learning process for the first time enabled UK housing policy-makers and other stakeholders who participated in the project to move beyond a decision-making method focusing on single-objective policies (for example reducing the carbon footprint of the housing stock, or addressing fuel poverty) and unintended consequences, towards decision-making that considers what the shared objectives are for policies about housing and identifies more effective policy levers that could optimise those shared objectives. By the end of a second workshop, representatives had begun to discuss policy options and their (often conflicting) short- and long-term dynamic implications using the CLDs, demonstrating the utility of this collaborative learning approach, as well as revealing shifts in thinking as a result of participation. Understanding housing, energy and wellbeing as a complex system is an important first step in being able to identify more effective policy levers, in contrast to the current collection of disparate information, which fails to support effective assements of policy options.

### Limitations

The CLDs alone have limited validity, since they currently reflect the collective knowledge of stakeholders combined with some initial literature review. Nevertheless, they represent an improvement on current practices of decision-making for UK housing. A great deal of further work is needed to test the agreed and disputed relationships by bringing together the best available data and research, aiming for a model that supports reflection and exploration of options rather than point prediction.

The validity and robustness of participatory system dynamics models and the collaborative learning process depends heavily on including an appropriate mix of stakeholders in the process to achieve a causal diagram that is as comprehensive and accurate as possible [5, 41]. One of the strengths of this research has been the level of commitment across government, community, industry and academic stakeholders. However, participation is lacking in some areas. Within government, the Treasury is a powerful actor in policy-making about housing, particularly at a time when property prices and turnover are seen by the government as a core driver of economic growth. Despite being nominally involved, Treasury representatives have thus far been absent in the modelling process. Furthermore, some important community organisations have also been missing so far; these include tenancy and homeowner associations, the National Housing Federation and organisations representing the homeless. Further work is currently underway to engage these agencies in the ongoing research.

### Implications for policy and research

By the time a working set of CLDs had been refined and discussed, stakeholders proposed some early policy insights and recommendations. It was suggested that successful decarbonisation of the UK housing stock requires the rapid establishment of a cross-government group to develop meaningful systems thinking capacity. This group would need to be supported by an advisory committee. The importance of local social connection in the minds of stakeholders suggests that policies should support the strengthening of community capacity to drive change. A number of parts of the overall map suggest that improving tenure security in the private rental sector would strengthen a number of beneficial feedback loops for wellbeing and decarbonisation. However, the assumption that mixed tenure types leads to greater community connection needs testing. Greater cross-government consensus about objectives in the national property market would enable further work to understand effective policies that would have benefits across a range of wellbeing and energy outcomes.

Priorities for future research were also suggested. An existing energy or housing policy could be used to consider the theoretical relationships identified in the causal loop diagrams. There was a lack of feedback loops identified in the area of housing energy efficiency. It was suggested that this requires further investigation. Simulation of the adaptation to climate change feedback loops would allow policy makers to understand how important the reinforcing loops are in this diagram by demonstrating dynamically the energy and land costs of adaptation, compared with expected energy savings from energy efficiency improvements. Simulation of the fuel poverty and temperature optimisation loops would demonstrate whether the balancing or reinforcing loops are most likely to dominate as a result of future climate change for the housing stock. Development of widely agreed metrics to describe “quality” as it relates to both houses and neighbourhoods is also needed.

The qualitative modelling is the initial part of a larger piece of work. We are evaluating the effectiveness of participatory SDM in this context in keeping with current models of evaluation for transdisciplinary research [42]. We are using a combination of process and outcome evaluation that includes reflective review by stakeholders and researchers; more formal evaluation of usefulness; assessing changes in the discourse used during workshop policy discussions; and considering whether there have been changes in the policies considered effective. Levels of consensus across stakeholder groups about policy priorities and shifts in government policy will also be reported over time.

Strategic small pieces of simulation modelling will enable agreed and disputed relationships to be tested and refined in a collaborative learning environment. Simulation modelling will be critical for understanding the comparative strengths of different feedback loops, as well as their changing behaviour over time to support improved decision-making. The culmination of this iterative process of simulation and refinement should be the simulation of realistic policy options to assess their dynamic future effects on the shared policy criteria.

Bringing together the results of the participatory system dynamics modelling with multi-criteria decision analysis would allow stakeholders to more explicitly value outcomes and weigh up policy options.

## Conclusions

We have developed a comprehensive system model linking housing, energy and public health, with immediate usefulness for all those with a stake in housing policy in the UK. Furthermore, we have demonstrated the usefulness of participatory SDM as a collaborative learning process to support improved policymaking for housing that is able to integrate a broad range of outcomes across wellbeing, social and health equity, and environmental sustainability. Further work is needed to validate the model, include simulations to explore future policy options and combine SDM with other policy assessment tools, as well as methods to support shifts in the conceptual frameworks underpinning policy, that will be necessary for healthier more sustainable housing.

## Additional files

Additional file 1: Table S1. Map of participation across the qualitative model development (The number of stars denotes the number of participants. Grey stars show where new participants were added). (DOCX 54 kb) Additional file 2:
**Peer review reports.** (PDF 105 kb)

## Abbreviations

CLD: Causal loop diagram
GHGs: Greenhouse gas emissions
SDM: System dynamics modelling
UK: United Kingdom
