# Supplementary material for: Integrated decision-making about housing, energy and wellbeing: a qualitative system dynamics model
Source: Environ Health. 2016 Mar 8;15(Suppl 1):37. doi: 10.1186/s12940-016-0098-z (PMC4895316; doi:10.1186/s12940-016-0098-z)
Supplement: Additional file 1: Table S1. — Map of participation across the qualitative model development (The number of stars denotes the number of participants. Grey stars show where new participants were added). (DOCX 54 kb) [file 12940_2016_98_MOESM1_ESM.docx]

| Participating organisations | | Interviews | Workshop 1 | Workshop 2 | Other in depth input |
| --- | --- | --- | --- | --- | --- |
| National government departments | Business, Innovation and Skills | ★ |  | ★ |  |
|  | Communities and Local Government | ★★ |  | ★★ |  |
|  | Energy and Climate Change | ★★★ | ★★ | ★ |  |
|  | Environment, Food and Rural Affairs | ★ | ★ |  |  |
|  | Health | ★ | ★ |  |  |
|  | Public Health England | ★ | ★ | ★ |  |
| Local government | Core Cities group | ★★ | ★★ |  |  |
|  | Greater London Authority | ★ | ★ | ★★ |  |
|  | Sheffield City Council | ★★ | ★ |  |  |
| Non-government organisations and community members | Age UK | ★ | ★ |  |  |
|  | Community Roots Group |  |  | ★★★★ | ★★★★★★ |
|  | English Heritage |  | ★ | ★ | ★ |
|  | Fuel Poverty Advisory Group/ Consumer Focus | ★ | ★ | ★★ |  |
|  | Individual buildings research expert | ★ | ★ | ★ | ★ |
|  | New Economics Foundation | ★ |  | ★ |  |
|  | Positive Money | ★ | ★ | ★ | ★ |
|  | Shelter | ★ |  |  |  |
|  | Sustainable by Design |  | ★ | ★ |  |
|  | Sustainable Development Foundation | ★ | ★ | ★ | ★ |
|  | Usable Buildings Trust | ★ |  |  |  |
|  | Zero Carbon Hub | ★ |  | ★ |  |
| Industry | AECOM |  |  | ★ | ★★★★ |
|  | Affinity Sutton | ★ | ★ |  |  |
|  | Arup Consultancy | ★ | ★★★ | ★ |  |
|  | Chartered Institute of Buildings Services Engineers | ★ | ★ | ★ |  |
|  | EDF Energy |  | ★ | ★ |  |
|  | Good Homes Alliance | ★ | ★ |  |  |
|  | Homebuilders Federation | ★ |  |  |  |
|  | Parity Projects | ★ |  |  |  |
|  | Wilmott Dixon |  | ★ |  |  |
| Academic departments | Adaption and Resilience to Climate Change UKCIP, University of Oxford | ★ |  | ★ |  |
|  | Centre for Regional Economic and Social Research, Sheffield Hallam | ★ | ★★ |  |  |
|  | Centre for Sustainable Planning and Environments, University of the West of England | ★ | ★ |  |  |
|  | Dynamics of Energy, Mobility and Demand Centre, Department of Sociology, Lancaster University | ★★ |  |  |  |
|  | Environmental Change Institute, University of Oxford |  |  |  | ★ |
|  | Leeds Sustainability Institute, Leeds Metropolitan | ★ |  | ★ | ★ |

Supplemental Table 1 Map of participation across the qualitative model development (The number of stars denotes the number of participants. Grey stars show where new participants were added)
