# Supplementary material for: Integrated decision-making about housing, energy and wellbeing: a qualitative system dynamics model
Source: Environ Health. 2016 Mar 8;15(Suppl 1):37. doi: 10.1186/s12940-016-0098-z (PMC4895316; doi:10.1186/s12940-016-0098-z)
Supplement: Additional file 2: — Peer review reports. (PDF 105 kb) [file 12940_2016_98_MOESM2_ESM.pdf]

Reviewer 1

Dear Sotiris,

Please find my review of the submitted paper below.

The paper "Integrated decision-making about housing, energy and wellbeing: a qualitative system dynamics model" provides an account of a large-scale group model building project in the UK. It seeks to improve stakeholder awareness of the interconnections between housing, energy preservation and social and physical well-being in the UK housing stock. Its main methodology is group model building or qualitative system dynamics, using causal diagrams in order to clarify the problem structure and to improve common understanding in multi-actor settings. The method is appropriate as it has repeatedly and empirically demonstrated positive effects on problem understanding. Furthermore, the method has proven helpful in numerous cases for improving and innovating policy logic.

The relevance of the project theme, i.e. speeding up energy saving in the housing stock while simultaneously safeguarding other interests, is self-evident. What makes this project unique and an outstanding piece of work is the scale of stakeholder involvement in a structured process of causal diagram creation and validation. The average group model building project encompassed five to ten participants from three or four organizations or departments. This project has successfully managed to work with more than 50 participants from 37 widely varying organizations in several sessions. The project has yielded seven thematically different causal loop diagrams on interconnected subthemes, of which one is presented in a certain amount of detail. The paper furthermore presents the steps ahead in this remarkable project, such as further improvements of the causal structures and translation of the diagrams into quantitative simulation models for policy simulation.

The paper as such is well-written and provides a clear account of the stakeholder participation process. On the other hand, the detailed explanation of only one substructure leaves me longing for more. I would really welcome to see more diagrams explained, so that readers can grasp the magnitude of the work done. For the phase of model quantification, the researchers will certainly face challenges as there are several well-documented pitfalls in the translation of causal loop diagrams into quantitative models. I am certain that the system dynamics community is willing to help out with these challenges. The relevance of the problem and the effort put in the phase of stakeholder involvement more than justify far reaching support. Furthermore, I suspect and hope that projects like these, strongly focusing on policy innovation can also help the use system dynamics in environmental issues to evolve beyond the well-known but somewhat fatalistic 1970's narrative of resource exhaustion into a more contemporaneous approach for innovating policies of climate change adaptation and mitigation.

Martijn Eskinasi PhD
PhD in system dynamics application in policy assesment

Reviewer 2

Integrated decision-making about housing, energy and wellbeing: a qualitative system dynamics model

Alexandra Macmillan, Michael Davies, Clive Shrubsole, Naomi Luxford, Neil May, Lai Fong Chiu, Evelina Trutnevyte, Yekatherina Bobrova, Zaid Chalabi.

Comments from reviewer 2: Paul Wilkinson

This is a very useful contribution to literature and should be published. It described the process of stakeholder participation to develop a more thorough understanding of the complex nature of policy influences as they relate to the housing sector in relation to human well-being. Its principal value is in describing the authors' experience of implementing an engagement process and the lessons learned from it.

Although the text is generally well-written, it would benefit from further clarification of the steps involved in their work, as well as some conceptual clarification of the ultimate objective of SDM, the role of quantitative modelling and the evaluation of its utility.

(1) It is important to be clear about terminology and definitions, and specifically what the authors mean by system dynamics modelling! The focus of the paper is very much on the elicitation of views on the interrelationships in a complex system in qualitative terms, yet there is also reference to quantitative modelling without being explicit as to whether this is an intrinsic part of the process and its ultimate objective. Many readers are likely to assume that SDM entails quantitative modelling. In the *Methods* section, there is sudden leap (top of page 4) to describing SD simulation as consisting of "a set of differential equations", which is surprising given the foregoing text and the main thrust of the rest of the paper which is about stakeholder engagement. It would be helpful to spell out clearly what SDM entails: development of a mathematical model, a qualitative process or both, and if so what the steps leading to the mathematical model are.

(2) I found it difficult to follow all the stages of the processes described in this work, and the interrelationship between them -- cognitive mapping, thematic analyses (including of secondary data), causal loop diagrams, assessment criteria, indictors etc. It would be very helpful to have a flow diagram showing all the steps in the process with clear and precise explanation, including of the how these stages relate to specific stakeholder interviews and workshops. Some specific points of note:
-- I was unclear about the reference to *sets* of causal diagrams, suggesting that a wider map of interconnections (implied by the focus on complex systems) is somehow disaggregated into component CLDs. Could this be clarified, please.
-- The last paragraph of page 6 refers to "the list of assessment criteria elicited during the interviews". Unless I have missed it, these criteria have not been described in any of the preceding text (except very briefly in the abstract) and I was not clear how they would be applied.
-- I was also unclear about the use of secondary data. I interpret secondary data to mean the

results of the literature review, but neither the review itself nor its analysis are described in sufficient detail. Could some further explanation be given of the numbers in Table 2, for example, and specifically of the meaning of ‘prevalence across the dataset’ (what is the dataset, what does prevalence represent).
-- I did not fully understand all of the connections of Figure 2. For example, the ‘balancing loop’ B2 seems not to relate to a loop at all (the arrows of the surrounding pathways don’t all go in the same direction).

(3) The interconnections elicited from the qualitative work in the causal loop diagrams are multiple and complex (as expected for a ‘complex system’), especially if all the components are put together. If there is an assumption that this evidence will eventually be translated into a quantitative model, it raises questions about the feasibility and stability of such models containing multiple feedback loops. Is it expected that such quantitative models are needed and that they provide accurate, or at least useful, representations of the real world? The application of quantitative models is implied by the discussion in the section on the *Implications for policy research* (page 13), in which there are various references to simulation of feedback loops as useful next steps for research to “allow policymakers to understand how important the reinforcing loops are” etc. Does this not depend upon quite detailed (quantitative) parameterization of the interconnections? If so, how would those parameters identified?

(4) How is the value of this SDM process to be judged? The processes described bring together multiple stakeholders, which it is reasonable to assume yields wider perspectives on the interconnections of housing, energy and well-being. But from a research perspective it is important to evaluate and document through rigorous methods what SDM achieves, by what criteria it leads to improve policy-making, and how such improvement can be objectively measured. Improved policy-making seems very likely, but this should not be assumed as the automatic consequence simply because more connections are mapped and a wider group of people is engaged. It would be useful to add some brief text on this.
